# 2-Hydrazinyl-4-methyl-1,3-benzothia­zole

**DOI:** 10.1107/S1600536811020149

**Published:** 2011-06-11

**Authors:** Xu-Feng Liu, Xiao-Yong Yu, Shao-Liang Jiang

**Affiliations:** a Department of Chemical Engineering, Ningbo University of Technology, Ningbo 315016, People’s Republic of China; b College of Pharmaceutical Science, Zhejiang University of Technology, Hangzhou 310014, People’s Republic of China

## Abstract

The title compound, C_8_H_9_N_3_S, is almost planar (r.m.s. deviation = 0.019 Å) apart from the terminal –NH_2_ grouping [deviation of the N atom = 0.286 (2) Å]. In the crystal, mol­ecules are linked by N—H⋯N hydrogen bonds, generating (001) sheets.

## Related literature

For related structures and their biactivity, see Sun & Cui (2008 ▶); Liu & Liu (2011 ▶); Liu*et al.* (2011*a* ▶,*b* ▶). For the synthesis, see: Patel *et al.* (2010 ▶).
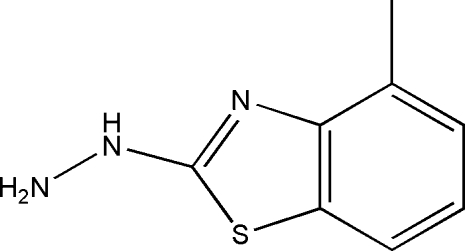


## Experimental

### 

#### Crystal data

C_8_H_9_N_3_S
*M* *_r_* = 179.24
Monoclinic, 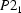

*a* = 3.893 (2) Å
*b* = 7.312 (4) Å
*c* = 14.137 (8) Å
β = 93.416 (13)°
*V* = 401.7 (4) Å^3^
*Z* = 2
Mo *K*α radiation
μ = 0.34 mm^−1^
*T* = 113 K
0.28 × 0.18 × 0.10 mm

#### Data collection

Rigaku Saturn CCD area-detector diffractometer
Absorption correction: multi-scan (*CrystalClear*; Rigaku/MSC, 2005 ▶) *T*_min_ = 0.910, *T*_max_ = 0.967
4186 measured reflections
1864 independent reflections
1614 reflections with *I* > 2σ(*I*)
*R*_int_ = 0.043

#### Refinement

*R*[*F* ^2^ > 2σ(*F* ^2^)] = 0.028
*wR*(*F* ^2^) = 0.057
*S* = 1.02
1864 reflections
122 parameters
5 restraints
H atoms treated by a mixture of independent and constrained refinement
Δρ_max_ = 0.28 e Å^−3^
Δρ_min_ = −0.20 e Å^−3^
Absolute structure: Flack (1983 ▶), 836 Friedel pairs
Flack parameter: −0.09 (6)

### 

Data collection: *CrystalClear* (Rigaku/MSC, 2005 ▶); cell refinement: *CrystalClear*; data reduction: *CrystalClear*; program(s) used to solve structure: *SHELXS97* (Sheldrick, 2008 ▶); program(s) used to refine structure: *SHELXL97* (Sheldrick, 2008 ▶); molecular graphics: *SHELXTL* (Sheldrick, 2008 ▶); software used to prepare material for publication: *SHELXTL*.

## Supplementary Material

Crystal structure: contains datablock(s) global, I. DOI: 10.1107/S1600536811020149/hb5894sup1.cif

Structure factors: contains datablock(s) I. DOI: 10.1107/S1600536811020149/hb5894Isup2.hkl

Supplementary material file. DOI: 10.1107/S1600536811020149/hb5894Isup3.cml

Additional supplementary materials:  crystallographic information; 3D view; checkCIF report

## Figures and Tables

**Table 1:** Hydrogen-bond geometry (Å, °)

*D*—H⋯*A*	*D*—H	H⋯*A*	*D*⋯*A*	*D*—H⋯*A*
N2—H2⋯N3^i^	0.89 (1)	2.30 (2)	2.996 (3)	135 (2)
N3—H3*A*⋯N1^ii^	0.92 (1)	2.21 (1)	3.077 (3)	156 (2)

Symmetry codes: (i) 
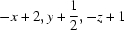
; (ii) 
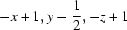
.

**Fig. 1.**
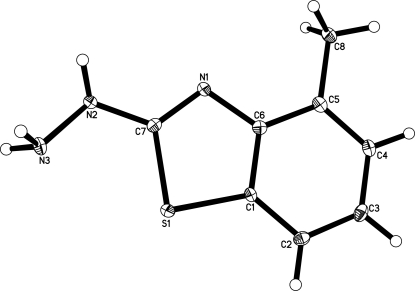
The molecular structure of (I). Displacement ellipsoids are drawn at the 30% probability level and H atoms are shown as small spheres of arbitrary radii.

**Fig. 2.**
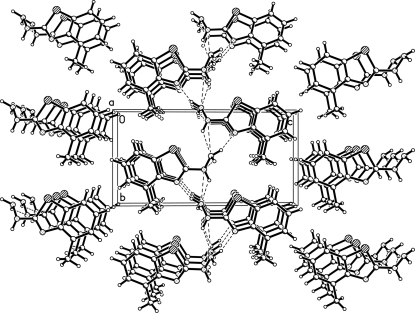
The crystal packing for (I).

## supplementary crystallographic information

### Comment

Sulfur and nitrogen heterocyclic compounds have received considerable attention in recent years because of their medicinal and pesticidal importance, such as 1,3,4-thiadiazoles, pyrimidines, and 1,2,4-triazoles (Liu & Liu, 2011; Liu *et al.*, 2011a,b). For a related structure, see Sun & Cui (2008).

Single-crystal X-ray diffraction analysis reveals that the title compound crystallizes in the monoclinic space group P2(1). As shown in Fig. 1, the benzene and thiazole rings are almost in the same plane. As shown in Fig. 2, there are intermolecular N—H···N hydrogen bonds in the crystal.

### Experimental

The title compound was prepared according to the literature procedures (Patel *et al.*, (2010). Colourless prisms of (I) were grown by the slow evaporation of an ethanol solution at room temperature.

### Refinement

All the H atoms were positioned geometrically (C—H = 0.93–0.97 Å) and refined as riding with *U*_iso_(H) = 1.2*U*_eq_(C) or 1.5*U*_eq_(methyl C).

### Crystal data

**Table:**

C_8_H_9_N_3_S	*F*(000) = 188
*M**_r_* = 179.24	*D*_x_ = 1.482 Mg m^−^^3^
Monoclinic, *P*2_1_	Mo *K*α radiation, λ = 0.71073 Å
*a* = 3.893 (2) Å	Cell parameters from 1356 reflections
*b* = 7.312 (4) Å	θ = 2.9–27.9°
*c* = 14.137 (8) Å	µ = 0.34 mm^−^^1^
β = 93.416 (13)°	*T* = 113 K
*V* = 401.7 (4) Å^3^	Prism, colorless
*Z* = 2	0.28 × 0.18 × 0.10 mm

### Data collection

**Table:**

Rigaku Saturn CCD area-detector diffractometer	1864 independent reflections
Radiation source: rotating anode	1614 reflections with *I* > 2σ(*I*)
multilayer	*R*_int_ = 0.043
Detector resolution: 14.63 pixels mm^-1^	θ_max_ = 27.9°, θ_min_ = 1.4°
ω and φ scans	*h* = −5→5
Absorption correction: multi-scan (*CrystalClear*; Rigaku/MSC, 2005)	*k* = −9→9
*T*_min_ = 0.910, *T*_max_ = 0.967	*l* = −18→18
4186 measured reflections	

### Refinement

**Table:**

Refinement on *F*^2^	Secondary atom site location: difference Fourier map
Least-squares matrix: full	Hydrogen site location: inferred from neighbouring sites
*R*[*F*^2^ > 2σ(*F*^2^)] = 0.028	H atoms treated by a mixture of independent and constrained refinement
*wR*(*F*^2^) = 0.057	*w* = 1/[σ^2^(*F*_o_^2^) + (0.006*P*)^2^] where *P* = (*F*_o_^2^ + 2*F*_c_^2^)/3
*S* = 1.02	(Δ/σ)_max_ < 0.001
1864 reflections	Δρ_max_ = 0.28 e Å^−^^3^
122 parameters	Δρ_min_ = −0.20 e Å^−^^3^
5 restraints	Absolute structure: Flack (1983), 836 Friedel pairs
Primary atom site location: structure-invariant direct methods	Flack parameter: −0.09 (6)

### Special details

**Table:**

Geometry. All e.s.d.'s (except the e.s.d. in the dihedral angle between two l.s. planes) are estimated using the full covariance matrix. The cell e.s.d.'s are taken into account individually in the estimation of e.s.d.'s in distances, angles and torsion angles; correlations between e.s.d.'s in cell parameters are only used when they are defined by crystal symmetry. An approximate (isotropic) treatment of cell e.s.d.'s is used for estimating e.s.d.'s involving l.s. planes.
Refinement. Refinement of *F*^2^ against ALL reflections. The weighted *R*-factor *wR* and goodness of fit *S* are based on *F*^2^, conventional *R*-factors *R* are based on *F*, with *F* set to zero for negative *F*^2^. The threshold expression of *F*^2^ > σ(*F*^2^) is used only for calculating *R*-factors(gt) *etc*. and is not relevant to the choice of reflections for refinement. *R*-factors based on *F*^2^ are statistically about twice as large as those based on *F*, and *R*- factors based on ALL data will be even larger.

### Fractional atomic coordinates and isotropic or equivalent isotropic displacement parameters (Å^2^)

**Table:**

	*x*	*y*	*z*	*U*_iso_*/*U*_eq_	
S1	0.74281 (13)	0.41481 (6)	0.33097 (3)	0.01462 (12)	
C5	0.2809 (5)	0.8047 (3)	0.18122 (14)	0.0144 (5)	
N3	0.9465 (5)	0.4807 (2)	0.52579 (12)	0.0163 (4)	
C2	0.5274 (5)	0.4513 (3)	0.13713 (13)	0.0163 (5)	
H2A	0.6107	0.3333	0.1221	0.020*	
N1	0.4899 (4)	0.7443 (2)	0.34709 (11)	0.0136 (4)	
C4	0.2524 (5)	0.7352 (3)	0.08962 (14)	0.0158 (5)	
H4	0.1480	0.8083	0.0403	0.019*	
C6	0.4388 (5)	0.6942 (3)	0.25178 (13)	0.0127 (4)	
C7	0.6412 (5)	0.6127 (3)	0.39382 (13)	0.0130 (4)	
N2	0.7110 (4)	0.6159 (2)	0.49033 (12)	0.0150 (4)	
C3	0.3711 (5)	0.5633 (3)	0.06809 (13)	0.0185 (5)	
H3	0.3451	0.5208	0.0046	0.022*	
C1	0.5572 (5)	0.5186 (3)	0.22906 (13)	0.0124 (4)	
C8	0.1482 (5)	0.9932 (3)	0.20484 (14)	0.0182 (5)	
H8A	0.3296	1.0618	0.2404	0.027*	
H8B	−0.0518	0.9812	0.2433	0.027*	
H8C	0.0810	1.0585	0.1461	0.027*	
H2	0.761 (6)	0.7236 (19)	0.5172 (16)	0.060 (10)*	
H3A	0.861 (4)	0.423 (3)	0.5774 (10)	0.043 (7)*	
H3B	1.153 (3)	0.531 (3)	0.5428 (12)	0.038 (8)*	

### Atomic displacement parameters (Å^2^)

**Table:**

	*U*^11^	*U*^22^	*U*^33^	*U*^12^	*U*^13^	*U*^23^
S1	0.0167 (3)	0.0120 (2)	0.0151 (2)	0.0012 (3)	0.00115 (18)	0.0003 (2)
C5	0.0078 (11)	0.0166 (11)	0.0191 (11)	−0.0007 (10)	0.0026 (9)	0.0030 (9)
N3	0.0141 (11)	0.0180 (10)	0.0163 (9)	0.0006 (8)	−0.0015 (7)	0.0049 (8)
C2	0.0142 (11)	0.0169 (14)	0.0180 (10)	−0.0005 (9)	0.0029 (8)	−0.0029 (9)
N1	0.0139 (10)	0.0126 (9)	0.0142 (9)	0.0000 (7)	0.0007 (7)	0.0017 (7)
C4	0.0124 (12)	0.0188 (11)	0.0163 (11)	−0.0008 (9)	0.0004 (8)	0.0044 (9)
C6	0.0091 (10)	0.0132 (10)	0.0160 (10)	−0.0037 (9)	0.0032 (8)	0.0004 (9)
C7	0.0125 (11)	0.0137 (10)	0.0133 (10)	−0.0043 (9)	0.0033 (9)	−0.0010 (9)
N2	0.0186 (11)	0.0117 (9)	0.0145 (9)	0.0011 (8)	−0.0009 (7)	0.0004 (8)
C3	0.0189 (13)	0.0231 (12)	0.0133 (10)	−0.0065 (9)	0.0009 (9)	−0.0015 (10)
C1	0.0113 (11)	0.0129 (11)	0.0129 (10)	0.0004 (9)	0.0005 (8)	0.0027 (8)
C8	0.0167 (13)	0.0162 (10)	0.0215 (11)	0.0010 (10)	−0.0008 (9)	0.0034 (9)

### Geometric parameters (Å, °)

**Table:**

S1—C1	1.746 (2)	N1—C7	1.289 (2)
S1—C7	1.756 (2)	N1—C6	1.399 (2)
C5—C4	1.389 (3)	C4—C3	1.379 (3)
C5—C6	1.397 (3)	C4—H4	0.9500
C5—C8	1.516 (3)	C6—C1	1.407 (2)
N3—N2	1.420 (2)	C7—N2	1.375 (3)
N3—H3A	0.923 (9)	N2—H2	0.891 (9)
N3—H3B	0.903 (9)	C3—H3	0.9500
C2—C3	1.387 (3)	C8—H8A	0.9800
C2—C1	1.388 (2)	C8—H8B	0.9800
C2—H2A	0.9500	C8—H8C	0.9800
			
C1—S1—C7	87.97 (10)	N1—C7—S1	117.74 (15)
C4—C5—C6	117.47 (18)	N2—C7—S1	118.61 (15)
C4—C5—C8	121.90 (18)	C7—N2—N3	115.08 (15)
C6—C5—C8	120.64 (17)	C7—N2—H2	117.6 (16)
N2—N3—H3A	110.0 (13)	N3—N2—H2	110.2 (17)
N2—N3—H3B	110.7 (14)	C4—C3—C2	121.44 (19)
H3A—N3—H3B	109.6 (12)	C4—C3—H3	119.3
C3—C2—C1	117.26 (17)	C2—C3—H3	119.3
C3—C2—H2A	121.4	C2—C1—C6	121.80 (18)
C1—C2—H2A	121.4	C2—C1—S1	128.72 (15)
C7—N1—C6	109.43 (16)	C6—C1—S1	109.47 (14)
C3—C4—C5	121.98 (19)	C5—C8—H8A	109.5
C3—C4—H4	119.0	C5—C8—H8B	109.5
C5—C4—H4	119.0	H8A—C8—H8B	109.5
C5—C6—N1	124.57 (18)	C5—C8—H8C	109.5
C5—C6—C1	120.04 (17)	H8A—C8—H8C	109.5
N1—C6—C1	115.39 (17)	H8B—C8—H8C	109.5
N1—C7—N2	123.57 (18)		
			
C6—C5—C4—C3	0.6 (3)	N1—C7—N2—N3	−165.94 (18)
C8—C5—C4—C3	−179.61 (17)	S1—C7—N2—N3	17.4 (2)
C4—C5—C6—N1	179.46 (18)	C5—C4—C3—C2	−0.4 (3)
C8—C5—C6—N1	−0.3 (3)	C1—C2—C3—C4	0.5 (3)
C4—C5—C6—C1	−0.9 (3)	C3—C2—C1—C6	−0.9 (3)
C8—C5—C6—C1	179.27 (16)	C3—C2—C1—S1	−179.19 (14)
C7—N1—C6—C5	179.87 (19)	C5—C6—C1—C2	1.1 (3)
C7—N1—C6—C1	0.3 (2)	N1—C6—C1—C2	−179.26 (17)
C6—N1—C7—N2	−176.41 (18)	C5—C6—C1—S1	179.73 (15)
C6—N1—C7—S1	0.3 (2)	N1—C6—C1—S1	−0.6 (2)
C1—S1—C7—N1	−0.54 (17)	C7—S1—C1—C2	179.11 (18)
C1—S1—C7—N2	176.31 (16)	C7—S1—C1—C6	0.61 (14)

### Hydrogen-bond geometry (Å, °)

**Table:**

*D*—H···*A*	*D*—H	H···*A*	*D*···*A*	*D*—H···*A*
N2—H2···N3^i^	0.89 (1)	2.30 (2)	2.996 (3)	135 (2)
N3—H3A···N1^ii^	0.92 (1)	2.21 (1)	3.077 (3)	156.(2)

Symmetry codes: (i) −*x*+2, *y*+1/2, −*z*+1; (ii) −*x*+1, *y*−1/2, −*z*+1.

## References

[bb1] Flack, H. D. (1983). *Acta Cryst.* A**39**, 876–881.

[bb2] Liu, X.-F. & Liu, X.-H. (2011). *Acta Cryst.* E**67**, o202.

[bb3] Liu, X. H., Tan, C. X. & Weng, J. Q. (2011*a*). *Phosphorus Sulfur Silicon Relat. Elem.* **186**, 552–557.

[bb4] Liu, X. H., Tan, C. X. & Weng, J. Q. (2011*b*). *Phosphorus Sulfur Silicon Relat. Elem.* **186**, 558–564.

[bb5] Patel, N. B., Khan, I. H. & Rajani, A. D. (2010). *Eur. J. Med. Chem.* **45**, 4293–4299. 10.1016/j.ejmech.2010.06.031 20630629

[bb6] Rigaku/MSC (2005). *CrystalClear* Rigaku/MSC Inc. The Woodlands, Texas, USA.

[bb7] Sheldrick, G. M. (2008). *Acta Cryst.* A**64**, 112–122. 10.1107/S0108767307043930 18156677

[bb8] Sun, Y.-F. & Cui, Y.-P. (2008). *Acta Cryst.* E**64**, o690. 10.1107/S1600536808006193 PMC2961026 21202082

